# Antioxidant defence of colostrum and milk in consecutive lactations in sows

**DOI:** 10.1186/2046-0481-65-4

**Published:** 2012-03-19

**Authors:** Justyna Lipko-Przybylska, Marta Kankofer

**Affiliations:** 1 Department of Animal Biochemistry and Physiology, Faculty of Veterinary Medicine, University of Life Sciences in Lublin, Akademicka 12, Lublin 20-033, Poland

**Keywords:** Antioxidative enzymes, Antioxidative vitamins, Colostrum, Sows

## Abstract

**Background:**

Parturition is supposed to be related to oxidative stress, not only for the mother, but also for the newborn. Moreover, it is not clear whether consecutive pregnancies, parturitions, and lactations are similar to each other in regards to intensity of metabolic processes or differ from each other. The aim of the study was to compare dynamic changes of antioxidative parameters in colostrum and milk of sows taken during 72 h postpartum from animals in consecutive lactations. Activities of glutathione peroxidase (GSH-Px), glutathione transferase (GSH-Tr), and superoxide dismutase (SOD), and amount of vitamin A and C were measured. Healthy pregnant animals were divided into 4 groups according to the assessed lactation: A -1^st ^lactation (n = 10), B - 2^nd ^and 3^rd ^lactation (n = 7), C - 4^th ^and 5^th ^lactation (n = 11), D - 6^th ^- 8^th ^lactation (n = 8). The colostrum was sampled immediately after parturition and after 6, 12, 18 and 36 h while the milk was assessed at 72 h after parturition. Spectrophotometric methods were used for measurements.

**Results:**

The activity of antioxidative enzymes and the concentration of vitamin A increased with time postpartum. The concentration of vitamin C was the highest between the 18th and 36th h postpartum.

**Conclusions:**

Dynamic changes in the values of antioxidant parameters measured during the study showed that sows milk provides the highest concentration of antioxidants in the 2^nd ^and 3^rd ^and 4^th ^and 5^th ^lactation giving the best defence against reactive oxygen species to newborns and mammary glands.

## Background

Pregnancy is a period of constant oxidative stress for the dam [1]. Moreover, perinatal stress and the related changes in oxygen partial pressure, may result in the exposition of the newborn to an excess of reactive oxygen species (ROS) during pregnancy, parturition, and postpartum. This happens mainly due to parturition-related alterations in steroid and prostaglandin metabolism in the dam but also due to the change from interauterine to extrauterine environment and the beginning of lung breathing in the newborn [2,3]. An uncontrolled imbalance between the production and neutralization of ROS may cause oxidative stress. It may lead to serious consequences not only for the cell membrane, which can change its permeability, but also for the proper course of metabolic processes, which can be altered due to peroxidative damage of enzymatic proteins and the presence of the toxic products of the peroxidative damage to macromolecules [4]. Antioxidant systems located in all cells and biological fluids protect biologically important proteins and other macromolecules from the peroxidative damage caused by ROS [5].

Colostrogenesis starts before parturition and results in production of a thick yellow fluid named colostrum which then accumulates in mammary glands and is secreted by the sows for 2-3 days after parturition [6]. After cessation of the colostrum secretion, it is referred to as mature milk, which differs from colostrum by the concentration of proteins which is due to the decrease in the content of immunoglobulins which stop crossing enteral barrier after 48 h postpartum. Thanks to its composition and properties, which are species specific and adjusted to current needs, colostrum and milk are the best nutrient for newborns and also give them the necessary protection from the endogenous (eg. trypsin inhibitors in colostrum protect from breakdown of immunoglobulins) and exogenous (environmental microorganisms) factors appearing after parturition [7].

There is evidence of the presence of antioxidative factors in colostrum and milk mainly in cows [8] but the data on the activity of these factors within the postparturient period and the species specific characteristics are missing. Data obtained from the cow colostrum and the milk showed dynamic changes in total antioxidant capacity (TAC) within 7 days postpartum [9]. Albera and Kankofer [10] compared the activity of lactoperoxidase, lactoferrin, and ceruloplasmin between cows and sows.

Zhao et al. [11] examined the TAC of women's colostrum and the TAC of the umbilical blood of their neonates. The authors underlined the importance of high TAC values for the health of the neonate with regards to the protection against ROS damage.

It is known that antioxidative mechanisms contain enzymatic and non-enzymatic factors that play particular role in concert action of the whole system. They include: superoxide dismutase (SOD) which is an enzymatic antioxidant detected in both the colostrum and the milk. Its mechanism of reaction is to reduce and oxidize metal ions that belong to active centers and to cause the dismutation of hydrogen peroxide. Glutathione peroxidase (GSH-Px) degrades hydrogen peroxide in the presence of glutathione that is also in colostrum and milk [8]. Glutathione transferase (GSH-Tr), also an enzymatic antioxidant, is present in many isoforms, takes part in glutathione metabolism and cooperates with glutathione peroxidases and reductases. It is one of the most important enzymes in the metabolism of xenobiotics [12].

Vitamin C, which has strong reductive properties, belongs to non-enzymatic antioxidants. Vitamin A neutralizes lipid oxidation in cell membranes.

There is scarce data available in literature on antioxidant parameters in colostrum and in the milk of sows or on the profile of changes in their activity after parturition. The influence of consecutive lactations on the activity and characteristics of the antioxidative system in the colostrum and the milk has not been described either and no data exists about the comparison of consecutive physiological parturitions with regard to the intensity of oxidative stress. Due to the high importance of the quality of colostrum for the newborn, the study was conducted in order to describe the mutual correlations of changes in the antioxidant parameters in colostrum and milk of sows after parturition and also to compare them in consecutive lactations.

**Figure 1:**
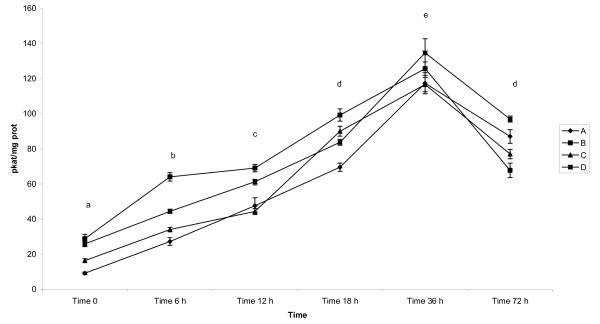
**Activity of GSH-Tr in colostrum and milk of sows after parturition**. Groups of animals: (A) - sows giving birth for 1^st ^time, (B) - sows giving birth for 2^nd^-3^rd ^time, (C) - sows giving birth for 4^th^-5^th ^time, (D) - sows giving birth for 6-8 times. GSH-Tr - Glutathione Transferase - mean - pkat/mg protein, GSH-Px - Glutathione Peroxidase - mean - nkat/mg protein, SOD - Superoxide Dismutase - mean - SOD units/mg protein, Vitamin C - mean - μmol/g protein, Vitamin A - mean - μg/g of protein. Time 0 - sample collected immediately after parturition, Time 6 h - sample collected 6 h after parturition, Time 12 h - sample collected 12 h after parturition, Time 18 h - sample collected 18 h after parturition, Time 36 - sample collected 36 h after parturition, Time 72 - sample collected 72 h after parturition Statistical significance describes the effect of time on antioxidative profile in colostrum and milk of sows during early postpartum period.

GSH-Tr activity (Table 1, Figure 1), expressed in pkat/mg protein (mean ± SD), in the colostrum of pigs was the lowest immediately after parturition in all examined lactations. It gradually significantly increased (p < 0.05) and reached its highest value 36 h after parturition. Afterwards, the activity decreased significantly (p < 0.05) in milk 72 h after parturition in all groups and reached similar value as found at 18 h after parturition.

**Table 1:** Effect of parity on antioxidative profile in colostrum and milk of sows during early postpartum period

Groups	GSH-Tr	GSH-Px	SOD	Vit C	Vit A
A	59.65^a^	36.01^a^	0.017^a^	0.34^a^	1.50^a^

B	75.76^c^	50.41^b^	0.021^b^	0.29^a^	2.28^c^

C	63.06^b^	52.08^b^	0.026^c^	0.30^a^	2.18^b, c^

D	74.48^c^	53.29^b^	0.020^b^	0.47^a^	1.98^b^

Groups of animals: (A) - sows giving birth for 1^st ^time, (B) - sows giving birth for 2^nd^-3^rd ^time, (C) - sows giving birth for 4^th^-5^th ^time, (D) - sows giving birth for 6-8 times

GSH-Tr - Glutathione Transferase - mean - pkat/mg protein, GSH-Px - Glutathione Peroxidase - mean - nkat/mg protein, SOD - Superoxide Dismutase - mean - SOD units/mg protein, Vitamin C - mean - μmol/g protein, Vitamin A - mean - μg/g of protein

Time 0 - sample collected immediately after parturition, Time 6 h - sample collected 6 h after parturition, Time 12 h - sample collected 12 h after parturition, Time 18 h - sample collected 18 h after parturition, Time 36 - sample collected 36 h after parturition, Time 72 - sample collected 72 h after parturition

Mean values differed significantly at p < 0.05 between examined groups (apart from pair B and D which did not differ significantly).

The interactions between time of sampling (F = 1186.19), the number of lactations (F = 72.75) as well as between each other (F = 19.06) were detected at p < 0.05, respectively.

**Figure 2:**
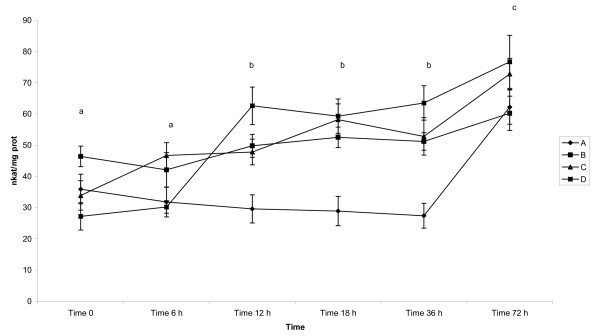
**Activity of GSH-Px in colostrum and milk of sows after parturition**. Groups of animals: (A) - sows giving birth for 1^st ^time, (B) - sows giving birth for 2^nd^-3^rd ^time, (C) - sows giving birth for 4^th^-5^th ^time, (D) - sows giving birth for 6-8 times. GSH-Tr - Glutathione Transferase - mean - pkat/mg protein, GSH-Px - Glutathione Peroxidase - mean - nkat/mg protein, SOD - Superoxide Dismutase - mean - SOD units/mg protein, Vitamin C - mean - μmol/g protein, Vitamin A - mean - μg/g of protein. Time 0 - sample collected immediately after parturition, Time 6 h - sample collected 6 h after parturition, Time 12 h - sample collected 12 h after parturition, Time 18 h - sample collected 18 h after parturition, Time 36 - sample collected 36 h after parturition, Time 72 - sample collected 72 h after parturition Statistical significance describes the effect of time on antioxidative profile in colostrum and milk of sows during early postpartum period.

GSH-Px activity (Table 1, Figure 2), expressed in nkat/mg protein (mean ± SD), in the colostrum of pigs was similar during first 6 h after parturition, increased significantly (p < 0.05) at 12 h and afterwards remained stable. Values in milk again increased significantly at p < 0.05 in all examined groups of animals.

Mean values in group A were significantly lower (p < 0.05) as compared to B, C and D which did not differ between each other.

The interactions between time of sampling (F = 52.62), the number of lactations (F = 13.92) as well as between each other (F = 8.44) were detected at p < 0.05, respectively.

**Figure 3:**
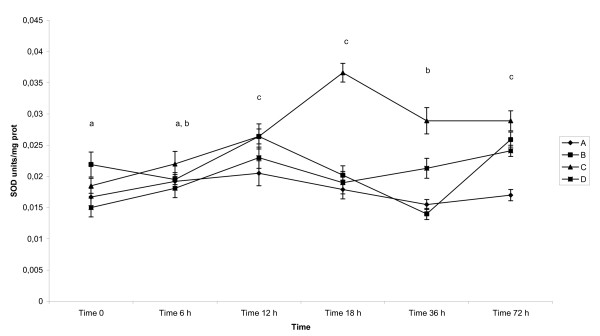
**Activity of SOD in colostrum and milk of sows after parturition**. Groups of animals: (A) - sows giving birth for 1^st ^time, (B) - sows giving birth for 2^nd^-3^rd ^time, (C) - sows giving birth for 4^th^-5^th ^time, (D) - sows giving birth for 6-8 times. GSH-Tr - Glutathione Transferase - mean - pkat/mg protein, GSH-Px - Glutathione Peroxidase - mean - nkat/mg protein, SOD - Superoxide Dismutase - mean - SOD units/mg protein, Vitamin C - mean - μmol/g protein, Vitamin A - mean - μg/g of protein. Time 0 - sample collected immediately after parturition, Time 6 h - sample collected 6 h after parturition, Time 12 h - sample collected 12 h after parturition, Time 18 h - sample collected 18 h after parturition, Time 36 - sample collected 36 h after parturition, Time 72 - sample collected 72 h after parturition Statistical significance describes the effect of time on antioxidative profile in colostrum and milk of sows during early postpartum period.

SOD activity (Table 1, Figure 3), expressed in SOD units/mg protein (mean ± SD), in the colostrum of sows fluctuated during examined period of time reaching the highest, significantly different (p < 0.05) values between 12-18 h as well as in milk at 72 h after parturition.

Mean values differed significantly (p < 0.05) between examined groups of animals apart from pair B and D which did not differ between each other.

The interactions between time of sampling (F = 23.87), the number of lactations (F = 57.08) as well as between each other (F = 14.82) were detected at p < 0.05, respectively.

**Figure 4:**
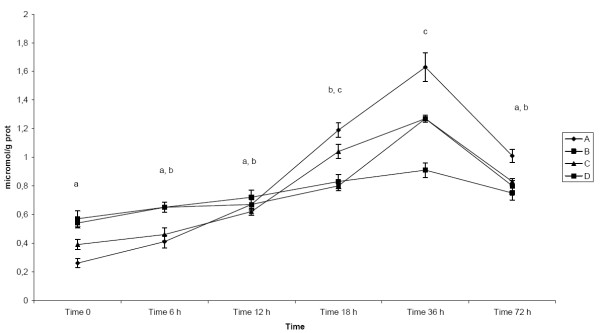
**Concentration of vitamin C in colostrum and milk of sows after parturition**. Groups of animals: (A) - sows giving birth for 1^st ^time, (B) - sows giving birth for 2^nd^-3^rd ^time, (C) - sows giving birth for 4^th^-5^th ^time, (D) - sows giving birth for 6-8 times. GSH-Tr - Glutathione Transferase - mean - pkat/mg protein, GSH-Px - Glutathione Peroxidase - mean - nkat/mg protein, SOD - Superoxide Dismutase - mean - SOD units/mg protein, Vitamin C - mean - μmol/g protein, Vitamin A - mean - μg/g of protein. Time 0 - sample collected immediately after parturition, Time 6 h - sample collected 6 h after parturition, Time 12 h - sample collected 12 h after parturition, Time 18 h - sample collected 18 h after parturition, Time 36 - sample collected 36 h after parturition, Time 72 - sample collected 72 h after parturition Statistical significance describes the effect of time on antioxidative profile in colostrum and milk of sows during early postpartum period.

The concentration of vitamin C (Table 1, Figure 4), expressed in μmol/g protein (mean ± SD), in the colostrum of sows showed increasing trend during examined period of time reaching the highest, significantly different (p < 0.05) values between 18-36 h after parturition and significant decrease (p < 0.05) in milk at 72 h after parturition.

Mean values showed no significant differences between groups of examined animals.

The interactions between time of sampling (F = 7.57) were detected at p < 0.05, respectively.

**Figure 5:**
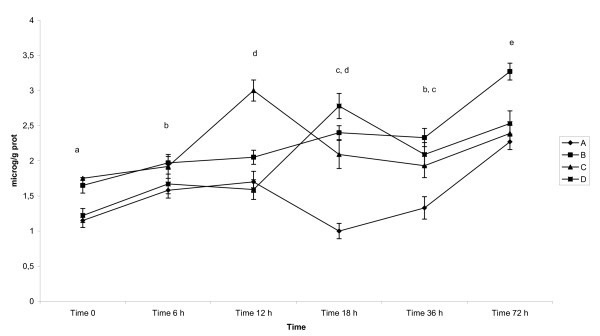
**Concentration of vitamin A in colostrum and milk of sows after parturition**. Groups of animals: (A) - sows giving birth for 1^st ^time, (B) - sows giving birth for 2^nd^-3^rd ^time, (C) - sows giving birth for 4^th^-5^th ^time, (D) - sows giving birth for 6-8 times. GSH-Tr - Glutathione Transferase - mean - pkat/mg protein, GSH-Px - Glutathione Peroxidase - mean - nkat/mg protein, SOD - Superoxide Dismutase - mean - SOD units/mg protein, Vitamin C - mean - μmol/g protein, Vitamin A - mean - μg/g of protein. Time 0 - sample collected immediately after parturition, Time 6 h - sample collected 6 h after parturition, Time 12 h - sample collected 12 h after parturition, Time 18 h - sample collected 18 h after parturition, Time 36 - sample collected 36 h after parturition, Time 72 - sample collected 72 h after parturition Statistical significance describes the effect of time on antioxidative profile in colostrum and milk of sows during early postpartum period.

The concentration of vitamin A (Table 1, Figure 5), expressed in μg/g of protein (mean ± SD), in the colostrum of pigs increased significantly (p < 0.05) up to 12 h after parturition, remained at similar level up to 36 h and again increased significantly (p < 0.05) in milk at 72 h after parturition.

Values differed significantly (p < 0.05) between examined groups of animals apart from pairs B and C as well as C and D which did not differ between each other.

The interactions between time of sampling (F = 53.07), the number of lactations (F = 37.61) as well as between each other (F = 14.70) were detected at p < 0.05, respectively.

## Discussion

The mammary gland undergoes glandular development, colostrogenesis, and lactogenesis at parturition. There are no data if this is age dependent or similar regardless of the pregnancy number. The regulation of the mammary gland growth and the milk production in prepartum sows depend on hormones while postpartum sows are stimulated by suckling and milk removal. Milk removal is related to (amongst other factors) the suckling interval, the piglet size, and the piglet behavior (see review [13]). The content of the sow colostrum and milk was previously described by Csapo et al. [6] but the data concerning the antioxidative defence in sow colostrum and milk are missing. Due to the fact that colostrum and milk are the only nutrients during the first days of life, their quality and composition should not only cover nutritive and immunological factors, but also the antioxidative demands of newborns to allow for their proper growth and development.

The analysis of the activity of antioxidant parameters in sows giving birth 1^st ^and several times that are presented here indicated generally higher values for 2-3 and 4-5 lactations in comparison to sows giving birth for the first or more than 5 times. These differences were evident, however the extent of differences was different for individual samples and for individual parameters. Moreover, the analysis of results showed dynamic changes in the profile of the antioxidative parameters within examined postpartal time within all groups of animals. The intensity of changes was, however, dependant on the number of lactations.

The SOD activity in the studied pigs increased until 12 - 18 h postpartum and remained at a similar level afterwards, which may indicate an adjustment of the antioxidant system to the current needs of the whole organism and the mammary gland in particular. The observed differences in the activity of the enzyme in relation to lactation may be connected with the intensification of stress symptoms during labour or with the need to reach a certain level of maturity in order to create adequate antioxidant defense. The activities of the other antioxidant enzymes showed an evident increase in time when compared with the onset of the parturition.

Studies of Snitynskyj et al. [14] showed a significant increase of SOD and GSH-Px activities in piglets' blood during the initial three days of life, whereas a similar increase of other enzymes (i.e. glucose-6-phoshate dehydrogenase or glutathione reductase) was noted between the 5^th ^and 10^th ^day of life. Ledwozyw and Kądziołka [15] achieved similar results, however these results concerned the activity of antioxidant enzymes in other organs. In their opinion, the highest activities of catalase, glutathione reductase, and SOD appeared in 2 day old piglets. Also the concentration of malondialdehyde - an indicator of the peroxidation intensity of lipids - was highest in the same animals. It confirmed the presence of a redox imbalance, however at the same time it confirmed an increase of antioxidant defence in the blood of newborn piglets and a need for additional colostrum defence, which increased dynamically from the parturition on.

Szczubial et al. [16] examined antioxidative status of periparturient sows. The studies on GSH-Px activity in the erythrocytes of sows were carried out 8 times during a period of 2 weeks before and 2 weeks after parturition. These results showed a significant decrease of activity 6-7 days before parturition and an increase 72 h before parturition, which lasted on the same level 2 weeks postpartum. It can be concluded that the organism of sow requires time to establish antioxidative/oxidative balance after pregnancy and parturition which is also reflected in colostrum and milk described in current study.

Correlations between carotene concentration and consecutive lactations are not well documented and mainly concern cows and calves. Some insignificant changes were described for cows who are primiparas, however no statistically significant changes were noted for cows who are multiparas. It was observed that the concentration of vitamin A in both colostrum and the milk of cows increased with age. It is probably connected with greater reserves of this vitamin in older animals, especially in the adipose tissue [17]. Similar conclusions may arise from our study.

Pig fetuses are able to synthesize vitamin C at the beginning of pregnancy. They lose it in advanced pregnancy due to a decrease in the activity of the synthesizing enzyme (L-gulono-gamma-lactone oxidase) [18,19]. Although Yen and Pond [20] compared the presence of placental transportation of vitamin C in piglets, the mechanism of this transportation still requires clarification. The authors stated that the concentration of vitamin C in fetal plasma at the end of pregnancy was a few-fold higher than in plasma of their mothers. It is probably connected with the transportation of this vitamin to the fetus and mammary gland. It is especially important, because pig newborns are not able to synthesize vitamin C in their first week of life and they depend on colostrum and milk as a source of it. A higher uptake of dehydroascorbic acid than of ascorbic acid was observed in placenta of women. Before birth, fetuses capture dehydroascorbic acid and transform it into ascorbic acid [21].

The presence of vitamin C in appropriate concentrations stimulates a better development of the skeletal system and collagen formation. Pinelli-Saavedra and Scaife [22] confirmed that the concentration of vitamin C is two times higher in colostrum than in milk, similar finds as in cows, and it increases dose-independently after supplementation. In the present studies the changes in the concentration of vitamin C in sows in consecutive samples and differences dependant on lactation were observed. Perhaps it should be regarded as achieving the appropriate reproductive age and being able to produce colostrum, which has the proper antioxidant potential.

The presence and activity of lactoperoxidase, lactoferrin, and ceruloplasmin (which also exert antioxidative properties) were described in colostrum and milk of sows by Albera and Kankofer [10]. While the ceruloplasmin antioxidative activity decreased within 36 h after parturition, the lactoferrin antioxidative activity increased and the lactoperoxidase fluctuated with a tendency to increase. The results confirm that postparturient time is characterized by dynamic changes in the antioxidative profile of colostrum and milk.

Studies on goats' colostrum carried out by Arguello et al. [23] did not show any influence of the consecutive lactation or the number of newborns in one litter on the physical properties of colostrum.

On the other hand, in cows evident (but not uniform) differences in the antioxidative/oxidative defense mechanisms seen between the first and second lactation were detected during early postparturient period [24].

Studies of Pinelli-Saavedra et al. [25] proved that it is possible to influence the content of both sow colostrum and milk by appropriate supplementation. However, the answers for questions if the sow is able to produce colostrum and milk in adequate quality and volume, as well as being similar during each lactation or if such supplementation is necessary, should be provided. Preliminary answers for it may come from current study.

## Conclusions

To conclude, studies on the antioxidant quality of colostrum and the milk of sows presented in this paper not only describe the dynamic changes of measured parameters during postnatal period, but also may indicate a possibility on how to adjust the antioxidant defence to the current needs of a newborn. What is more, the results may suggest a necessity to supplement pregnant sows in their first and further than fifth lactation, when the antioxidant defense seems to be weaker than in the second to fifth lactation periods.

## Materials and methods

### Experimental animals

The Local Ethics Committee, appointed at University of Life Sciences in Lublin, approved the experiment described below.

The studied animals (n = 36) were bred on a private farm in Cichawa in the Malopolska Region. Healthy, pregnant sows of Polish Landrace × Polish Large White, were kept on concrete grates with no bedding, only during the perinatal period was bedding provided. All sows included in the study were fed in the same way depending on the pregnancy time, with adequate fodder provided that was rich in minerals and vitamins necessary for pregnant and a lactating animal. Moreover, all sows had undisturbed postparturient period and similar number of piglets.

The animals were divided into 4 groups according to the studied pregnancy and lactation:

A. sows giving birth for the 1^st ^time (n = 10)

B. sows giving birth for the 2^nd^-3^rd ^time (n = 7)

C. sows giving birth for the 4^th^-5^th ^time (n = 11)

D. sows giving birth for 6^th ^- 8^th ^times (n = 8)

Colostrum (in volume of 6-7 ml) was sampled from clinically healthy animals by milking into clean plastic tubes always from the same bunch:

a) directly after parturition

b) 6 h after parturition

c) 12 h after parturition

d) 18 h after parturition

e) 36 h after parturition

f) 72 h after parturition - assumed to be mature milk

Directly after sampling, the colostrum and milk were portioned, frozen, and maintained in the temperature of -20°C until biochemical analysis.

The colostrum and milk for all measurements (except for vitamin A) were centrifuged over 10 min with the power of 2000 × g. The supernatant was used for analysis.

### Determination of GSH-tr activity - should be "Tr"

GSH-Tr catalyzes reactions between glutathione and 1-chloro-2,4-dinitrobenzene (CDNB); a coloured conjugate 2,4-dinitrophenyl-5-glutathione is created and measured spectrophotometrically at 340 nm [26]. The results were recalculated per protein concentration and given in pkat/mg protein.

### Determination of GSH-px activity - should be "Px"

The method is based on changes in absorbance connected with transformation of NADPH to NADP at 340 nm wavelength [27]. The results were recalculated per protein concentration and given in nkat/mg protein.

### Determination of SOD activity

The method is based on the measurement of percentage of inhibition for spontaneous auto-oxidation of adrenaline by SOD at 340 nm wavelength [28]. The percentage of inhibition was recalculated and given in SOD units/mg protein.

### Determination of vitamin C concentration

The method is based on the reaction with 2,6-dichlorophenolindophenol; the absorbance was measured at 520 nm wavelength. [29]. The results were given in μmol/g protein.

### Determination of vitamin A concentration

The method is based on spectrophotometric measurements of hexane layer at 325 nm [30]. The results were given in μg/g protein.

### Determination of protein concentration

Protein concentration was measured according to the biuret method [31] by use of ready kit (Cormay, Lublin, Poland). Recalculation of enzyme activities and concentrations of examined parameters per protein content allowed for a better comparison between colostrum and milk differing from each other by protein content.

### Statistical analysis

The observations (in duplicate) were averaged and subjected to statistical analysis of two-way ANOVA with multiple repeated measures (for non orthogonal data). Repeated measures ANOVA tests the equality of means. Longitudinal studies covering multiple sample collection within time from the same animals in matched groups were analysed with regard to possible interactions that may appear during consecutive lactations in examined parameters. Interactions between time of sampling after parturition, the number of lactations, and between these two main effects were calculated.

For further analysis of means HSD the Tukey test was implemented. A p value < 0.05 was considered as significant. Programme Statistica 6.0 (Statsoft) was used for the analysis. All results are presented as mean values.

## Abbreviations

GSH-Px: Glutathione peroxidase; GSH-Tr: Glutathione transferase; SOD: Superoxide dismutase; ROS: Reactive oxygen species; TAC: Total antioxidant capacity; CDNB: 1-chloro-2,4-dinitrobenzene.

## Competing interests

The authors declare that they have no competing interests.

## Authors' contributions

Both authors contributed equally to the conception and study design as well as analysis and interpretation of data. JP was more involved in analyzing data while MK in critical revising the manuscript. All authors read and approved the final manuscript.
